# Disrupted Functional Connectivity of Basal Ganglia across Tremor-Dominant and Akinetic/Rigid-Dominant Parkinson’s Disease

**DOI:** 10.3389/fnagi.2017.00360

**Published:** 2017-11-02

**Authors:** Xiaojun Guan, Qiaoling Zeng, Tao Guo, Jiaqiu Wang, Min Xuan, Quanquan Gu, Tao Wang, Peiyu Huang, Xiaojun Xu, Minming Zhang

**Affiliations:** ^1^Department of Radiology, The Second Affiliated Hospital, Zhejiang University School of Medicine, Hangzhou, China; ^2^Department of Neurology, The Second Affiliated Hospital, Zhejiang University School of Medicine, Hangzhou, China

**Keywords:** Parkinson’s disease, tremor-dominant, akinesia/rigidity-dominant, functional connectivity, functional magnetic resonance imaging

## Abstract

It is well known that disruption of basal ganglia function generates the motor symptoms in PD, however, these are presented in a heterogeneous manner; patients can be divided into tremor-dominant and akinesia/rigidity-dominant subtypes. To date, it is unknown if these differences in the motor symptoms could be explained by differences on the functional connectivity of basal ganglia with specific brain regions. In this study, we aimed to explore the alterations of the network-based and global functional connectivity linking to basal ganglia between the PD-TD and PD-AR patients. One hundred and six PD patients and 52 normal controls were recruited. According to the subscales of UPDRS motor scale, PD patients were divided into the PD-TD (*n* = 57) and PD-AR (*n* = 49) subtypes. We performed independent component analysis to identify basal ganglia network (BGN) involving connected brain regions having coactivation with basal ganglia. Eigenvector centrality mapping were processed and the eigenvector centrality in the subcortical component of BGN including the bilateral caudate nuclei, putamen, thalami and pallidum were extracted to measure the global connectivity. Compared with controls, whole PD patients or PD subtypes showed decreases of functional connectivity within the subcortical component of BGN, e.g., thalamus, pallidum and putamen. Compared with controls, decreased functional connectivity of precuneus and amygdala with basal ganglia was observed in the PD-TD while that of occipital lobule and precuneus was observed in the PD-AR. Compared with the PD-TD, significantly decreased functional connectivity between occipital lobule and cerebellum posterior lobule and basal ganglia was observed in the PD-AR, and such connectivity had positive correlations with tremor and negative correlations with akinesia/rigidity. We also observed enhanced global connectivity in the caudate nucleus and thalamus in the PD subtypes compared with controls. In conclusion, PD patients independent of motor subtypes consistently express similar alterations of functional connectivity within the subcortical component of BGN including network-based connectivity and global connectivity. Functional connectivity of cerebellum posterior lobule and occipital lobule with basal ganglia play important roles in the modulation of parkinsonian motor symptoms.

## Introduction

Parkinson’s disease (PD) is one of the most common neurodegenerative diseases with a morbidity of 1.7% for those aged ≥65 years in China (Zhang et al., 2005). PD is not a homogenous disease for its complicated motor symptoms, such as tremor, akinesia and rigidity, which could be mainly divided into tremor-dominant patients (PD-TD) or akinesia/rigidity-dominant patients (PD-AR) (Kang et al., 2005; Zaidel et al., 2009). Clinical literatures record that PD-AR patients have a greater progression of motor scores compared with PD-TD (Post et al., 2007; Reinoso et al., 2015; Zhang et al., 2016). Growing evidence revealed that there are different neuronal pathological underpinnings in these two subtypes including structure (Luo et al., 2017; Piccinin et al., 2017), function (Lewis et al., 2011) and iron distribution (Jin et al., 2012; Guan et al., 2017). However, it is still far to understand the aberrant pathophysiology of PD-TD and PD-AR.

The loss of dopaminergic neurons in substantia nigra pars compacta is the hallmark of PD in clinical stage (Braak et al., 2003). Due to the depletion of dopamine, the secondary disruption of basal ganglia function plays a heart role in the generation of motor impairment (Obeso et al., 2000; Szewczyk-Krolikowski et al., 2014; Rolinski et al., 2016). However, the classical model of basal ganglia function (cortico-striatal-thalamic loop) cannot fully interpret the diverse motor symptoms, especially tremor (Obeso et al., 2000; Zaidel et al., 2009). The network connected with basal ganglia (BGN) is complicated for it is extensively connected with other brain regions (Ashby et al., 2010). Few study has been performed to clarify the notion that whether dysfunction of BGN is a common finding regardless of motor subtypes or differences exist between PD-TD and PD-AR patients. Taken together, we hypothesized that dysfunction of functional connectivity in BGN could be seen in whole PD group and the PD subtypes, while differences of functional connectivity connecting with basal ganglia would be observed between the PD-AR and PD-TD.

Methodologies of resting-state functional magnetic resonance imaging (MRI) (rsfMRI) allow researchers to investigate brain functional connectivity in PD from different views. Independent component analysis (ICA) is a data-driven blind source separation method for decomposing a multivariate signal into independent networks (Smith et al., 2009). By using spatial ICA on rsfMRI data, distinct functional components, such as BGN (Szewczyk-Krolikowski et al., 2014; Rolinski et al., 2016), could be extracted successfully. Therefore, brain regions coactivating with basal ganglia would be identified. Eigenvector centrality mapping (ECM) is a computationally efficient tool for capturing intrinsic neural architecture on a voxel-wise level (Lohmann et al., 2010). It could calculate the globally weighted connectivity of the subcortical component of BGN, e.g., caudate nucleus, putamen, pallidus and thalamus. Therefore, eigenvector centrality (EC) of these structures could be obtained.

In the present study, we recruited 106 PD patients (57 PD-TD/49 PD-AR patients) and 52 normal controls. We performed ICA to identify BGN involving connected brain regions having coactivation with basal ganglia. In addition, ECM images were processed and the EC within above subcortical regions were extracted. We aimed to explore the alterations of network-based and global functional connectivity linking to basal ganglia between the PD-TD and PD-AR patients, further to investigate the relationships of aberrant functional connectivity with different motor impairments (tremor and akinesia/rigidity).

## Materials and Methods

### Subjects and MRI Scanning

One hundred and sixty PD patients and 55 control subjects signed informed consent forms in accordance with the approval of the Medical Ethic Committee of Second Affiliated Hospital of Zhejiang University School of Medicine. Diagnosis of PD was made by an experienced neurologist according to UK Parkinson’s Disease Society Brain Bank criteria (Hughes et al., 1992). Motor scales of the Unified Parkinson’s Disease Rating Scale (UPDRS), the Mini-Mental State Examination (MMSE) and disease duration were obtained from all patients. For patients taking medication, clinical assessments and image scanning were carried out after withholding all anti-parkinsonian medicine overnight (at least 12 h).

Patients (n) and control subjects (m) were excluded if they had: (1) history of a head injury, psychiatric or neurological diseases (except PD for the patients) (*n* = 11 with atypical parkinsonism; *m* = 1 with cerebral infarction); (2) alcohol or drug dependency or abuse; (3) contraindications or metal artifact for MRI scanning (*n* = 2); (4) severe head motion over 2 mm or/and 2 degrees (*n* = 9 and *m* = 1, details in *Imaging processing* section); (5) poor imaging quality (*n* = 7 and *m* = 1); (6) incomplete clinical data (*n* = 4); (7) on medication (*n* = 1); (8) according to MMSE estimated by the criteria suitable for Chinese population (MMSE score ≤ 17 for illiterate subjects, ≤20 for grade-school literate, and ≤23 for junior high school and higher education literate) (Katzman et al., 1988; Zhang et al., 1990), 7 PD patients with cognitive impairment were excluded from present study.

According to the subscales of UPDRS motor scale excluding the postural instability and gait difficulty items, we calculated the tremor score and akinetic/rigid score from remaining patients, and then, the PD-TD and PD-AR patients were defined following previous study (Kang et al., 2005). For each patient, the mean UPDRS motor tremor score (sum of items 20 and 21 divided by 4) and the mean UPDRS motor akinetic/rigid score (sum of items 22–27 and 31 divided by 15) were obtained. Based on the ratio of tremor score to akinetic/rigid score (subtype ratio), PD patients were grouped into the PD-TD subtype if the ratio was >1.0 (*n* = 57); into the PD-AR if the ratio was <0.8 (*n* = 49); and into the mixed subtype if the ratio was between 0.8 and 1.0 (*n* = 13). For the mixed PD subtypes had no symptom preference and small sample size, we excluded the patients assigned to this group. Finally, 106 PD patients (42 patients were drug-naive) with an average age of 57.5 ± 10.1 years were divided into 57 PD-TD (58.7 ± 10.2 years) patients and 49 PD-AR (56.0 ± 9.9 years) patients and 52 normal controls (57.6 ± 10.8 years) were included in the present study. **Table 1** showed the demographic characteristics and disease condition of the participants.

**Table 1:** Demographic and basic head motion parameters of recruited subjects.

	Normal controls	PD-TD	PD-AR	*p*-values	PD	*p*-values
No.(male/female)	52 (29/23)	57(33/24)	49 (23/26)	0.498	106 (56/50)	0.728
Age, y, mean ± SD	57.6 ± 10.8	58.7 ± 10.2	56.0 ± 9.9	0.886	57.5 ± 10.1	0.943
Transformation, mm	0.06 ± 0.04	0.05 ± 0.02	0.06 ± 0.04	0.163^a^	0.05 ± 0.03	0.063^a^
Rotation, degree (10^-4^)	6.3 ± 2.7	6.7 ± 3.5	7.1 ± 3.1	0.499	6.9 ± 3.6	0.311
Disease duration, years, mean ± SD	–	4.8 ± 4.2	4.4 ± 4.0	0.804^a^	–	–
Hoehn-Yahr, mean ± SD	–	2.2 ± 0.6	2.3 ± 0.7	0.606^a^	–	–
UPDRS III scores, mean ± SD	–	29.0 ± 17.3	28.8 ± 14.8	0.983	–	–
Subtype ratio, mean ± SD	–	2.2 ± 1.3	0.33 ± 0.3	**<0.001**	–	–
Tremor scores, mean ± SD	–	8.3 ± 4.6	2.1 ± 2.1	**<0.001**^a^	–	–
Akinetic/rigid scores, mean ± SD	–	17.0 ± 11.4	21.4 ± 10.4	**0.041**	–	–
MMSE scores, mean ± SD	–	27.5 ± 2.3	27.6 ± 2.2	0.784	–	–

*^a^Kruskal–Wallis test or Mann–Whitey *U-*test. Bold: Significant difference.*

### MRI

All subjects were scanned in a 3.0 Tesla MRI machine (GE Medical Systems, Signa EXCITE, Milwaukee, WI, United States) equipped with an eight-channel head coil. During MRI scanning, the head of each subject was stabilized with restraining foam pads. Earplugs were provided to reduce the noise during scanning. rsfMRI images were acquired using a gradient recalled echo/echo planar imaging sequence: repetition time = 2,000 ms; echo time = 30 ms; flip angle = 90°; field of view = 240 mm × 240 mm; matrix = 64 × 64; slice thickness = 5 mm; slice gap = 1 mm; 23 interleaved slices. A total of 185 volumes were acquired from each subject.

### Imaging Preprocessing

Data preprocessing was performed using SPM8^1^ and the Data Processing and Analysis for (Resting-State) Brain Imaging, DPABI^2^ (Yan et al., 2016). Firstly, the first 10 time points of rsfMRI scans were discarded due to consideration of instability of the initial MRI signal, thus 175 time points were implemented into following procedures: for ECM, slice timing, realignment, nuisance covariates regression (Friston 24, white matter and cerebrospinal fluid signal), normalizing by using EPI templates, smoothing (with a Gaussian kernel of 6 mm × 6 mm × 6 mm full width at half maximum), temporal band-pass filtering (0.01–0.1 Hz), detrending and scrubbing (of note, given that the removal of global signal is associated with the emergence of negative correlations (Murphy et al., 2009) which are still difficult to interpret, we did not regress out the global signal in the present study); for ICA, the procedures mentioned above excluding nuisance covariates regression, temporal band-pass filtering, detrending and scrubbing were conducted. Averaged scan-to-scan head motion was calculated including mean transformation and mean rotation parameters.

### ICA

All processed data from the rsfMRI of the PD patients and controls were analyzed using a Group ICA toolbox (GroupICAT version 4.0a)^3^. Independent components (IC) estimation mainly included three steps: data reduction, application of the ICA algorithm and back-reconstruction. The data dimensionality was reduced using two steps of principal component analysis, and the optimal number of IC was estimated using the minimum description length algorithm (40 components were finally estimated). Then, the Informix algorithm was used to run the ICA (Bell and Sejnowski, 1995). Finally, the temporospatial back-reconstruction method was used to generate time courses and spatial maps for each participant.

### ECM

Eigenvector centrality mapping of the preprocessed data was performed using the fast ECM (fECM) tool^4^. It does so by counting both the number and quality of connections so that a node with few connections to some other high-ranking nodes may outrank one with a larger number of low-ranking connections. As with its success in the Web search engine, ECM has also been proven valuable in analyzing human brain networks. fECM tool is fast and computationally efficient because it computes matrix-vector products without having to compute or store the connectivity matrix (Wink et al., 2012). Several studies had confirmed its role in investigating network alterations in neurodegenerative diseases (Lou et al., 2015; Luo et al., 2016; Qiu et al., 2016) and smoking addiction brain (Shen et al., 2017).

### Subcortical Regions of Interest

For ROI-based analysis, we used WFU_Pickatlas toolbox^5^ to define each region within basal ganglia, including bilateral caudate nuclei, putamen and pallidum. As thalamus interconnects closely with basal ganglia, bilateral thalami were also defined as targeted regions. All of these regions were belonging to the subcortical component of BGN. Parameter estimates (PE) (network-based functional connectivity) and EC in these regions were extracted from ICA images and ECM images by using an automated tool Marsbar^6^, respectively.

### Statistics Analysis

All statistical analyses in this study excepting the voxel-wised analyses implemented in the DPABI were performed using IBM SPSS Statistics version 19.0. The normal distribution of data was confirmed using the one-sample Kolmogorov–Smirnov test. Tremor score, disease duration, Hoehn-Yahr stage and transformation parameter were not distributed normally (*p* < 0.05). Therefore, Kruskal–Wallis test and Mann–Whitey *U* test were conducted appropriately. Differences in the age, gender, UPDRS motor score, akinetic/rigid score, the ratio value, MMSE score and rotation parameter among three groups or between the two subtypes (PD-TD vs. PD-AR, PD vs. Controls) were appropriately compared by analysis of variance (ANOVA) or Pearson’s chi-square or independent samples *t*-test. The inter-group differences of the extracted PE and EC of each region in the subcortical component of BGN were performed using general linear model regarding age and gender as covariates. And head motion parameters were additionally added as covariates in analyzing ECM data. The Bonferroni method was used to correct for multiple comparisons in these ROI-based comparisons (*p* < 0.05 was regarded as significance).

One sample *t*-test was performed to identify the overview of BGN by using ICA (*p* < 0.05, FDR corrected). Voxel-wised analysis was used to compare the PE distribution among the PD-TD, PD-AR and controls. ANOVA was first used to identify differential BGN regions among the three groups. Age and gender were regarded as covariates. The threshold was set at single voxel *P* < 0.01 and cluster size > 32 voxels using AlphaSim for multiple comparison corrections. To reduce false-positive results, we used the same mask and criteria as those used in the ANOVA. In this way, the gray matter mask has much more voxels than the brain areas identified by the ANOVA. Signals from the significant clusters by comparing the PD-AR and PD-TD groups were also extracted to test the correlation between PE and motor impairments (tremor score and akinesia/rigidity score, respectively). Age and gender were regarded as covariates. Because of the significant correlation between tremor and akinetic/rigid scores (*r* = 0.391, *p* < 0.001), we regressed out one motor impairment during analyzing the other one. Of note, due to majority of PD patients showing both motor impairments, partial correlation analyses were conducted in whole PD group (*n* = 106).

In addition, in order to detect the alterations of BGN in early PD patients (Hohen-Yahr stage = 1.0/1.5, *n* = 16), the same statistical analyses were conducted between 16 early PD patients and 52 controls.

## Results

### Demographic and Clinical Data

No significant differences of age, gender and head motion parameters were observed among PD-TD, PD-AR and controls or between the PD subtypes or between whole PD and controls. Excepting for the subtype ratio (*p* < 0.001), tremor score (*p* < 0.001) and akinesia/rigid score (*p* = 0.041), there was no significant difference in disease duration (*p* = 0.804), Hoehn–Yahr stage (*p* = 0.606), UPDRS motor score (*p* = 0.983), and MMSE score (*p* = 0.784) between the PD subtypes. All of these data were shown in **Table 1**.

### Basal Ganglia Network Identified by ICA

**FIGURE 1:**
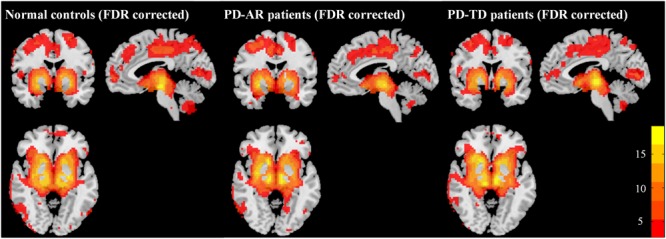
Patterns of basal ganglia network in PD-AR, PD-TD and normal controls (FDR-corrected, *p* < 0.05). One-sample *t*-test showed that all three groups showed similar pattern of basal ganglia network. PD-AR, Akinesia/rigidity-dominant Parkinson’s disease. PD-TD, Tremor-dominant Parkinson’s disease.

The structures in the basal ganglia are potential “hubs” connecting extensively with cortex and limbic system. Accordingly, in the present study, the so called “BGN” identified by ICA, could be explained as the coactivation between basal ganglia structures (bilateral caudate nulcei, putamen and pallidum) and other connected regions including bilateral thalami, medial superior frontal lobules, parietal lobules, occipital lobules and that in limbic system (e.g., bilateral cingulum, insula, amygdala, hippocampus and parahippocampus). In addition, we also observed coactivation in the cerebellum posterior lobule. One-sample *t*-test showed that all three groups had a similar BGN pattern (**Figure 1**).

### Inter-group Comparisons of ICA

**FIGURE 2:**
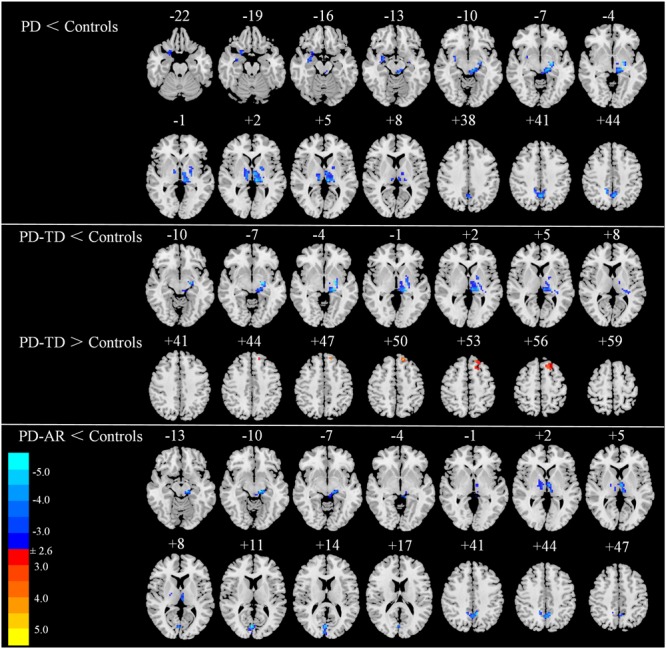
Voxel-wised comparisons of basal ganglia network between PD/PD subtypes and normal controls (*p* < 0.01, cluster size > 32, AlphaSim corrected).

**FIGURE 3:**
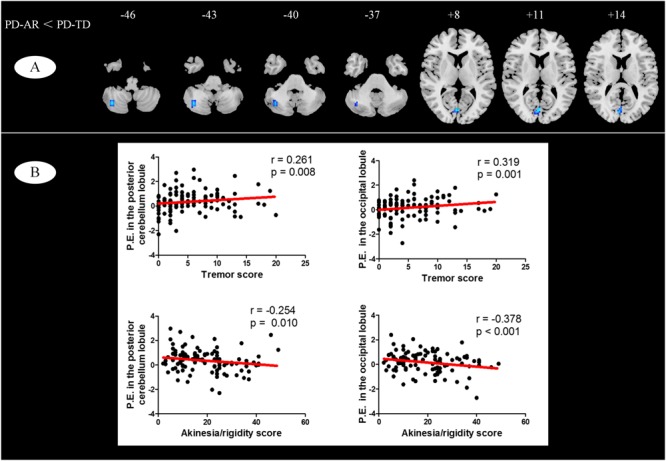
**(A)** Voxel-wised comparisons of basal ganglia network between PD-TD and PD-AR patients (*p* < 0.01, cluster size > 32, AlphaSim corrected); **(B)** Partial correlation analysis between parameter estimates measured by ICA algorithm and motor impairments (tremor or akinesia/rigidity).

In voxel-wised analysis (**Figure 2** and **Table 2**), we observed significant decreases of PE in the bilateral thalami extending to left putamen and left pallidum, bilateral precuneus and right amygdala in whole PD patients compared with controls. Compared with controls, the PD-AR patients showed significantly reduced PE in the bilateral thalami, occipital lobules and precuneus while the PD-TD patients showed reduced PE in the left thalamus which was extending to ipsilateral putamen and pallidum and increased PE in the left superior frontal lobule. More intriguingly, the PD-AR patients had significantly lower PE in the bilateral occipital lobules and right cerebellum posterior lobule than the PD-TD patients (**Figure 3A**).

**Table 2:** Differences of functional connectivity among PD-AR, PD-TD patients and controls.

			Peak MNI coordinate			
Brain Regions	L/R/B	Cluster size	X	Y	Z	Peak *T* value
**PD < controls**
Thalamus	R	42	9	–21	6	–3.56
Thalamus	L	228	–9	–24	3	–4.36
Precuneus	B	74	0	–57	42	–4.07
Amygdala	R	55	27	0	–12	–3.31
**PD-AR < controls**
Thalamus	B	124	–15	–24	–9	–4.12
Occipital lobule	B	43	3	–78	12	–4.00
Precuneus	B	47	–9	–51	42	–3.92
**PD-TD < controls**
Thalamus	L	185	–24	–12	–6	–4.65
**PD-TD > controls**
Superior Frontal lobule	L	44	–15	39	51	3.93
**PD-AR < PD-TD**
Posterior cerebellum lobule	R	42	36	–63	–45	–3.79
Occipital lobule	B	40	0	–75	12	–3.81

*L, Left; R, Right; B, Bilateral.*

**FIGURE 4:**
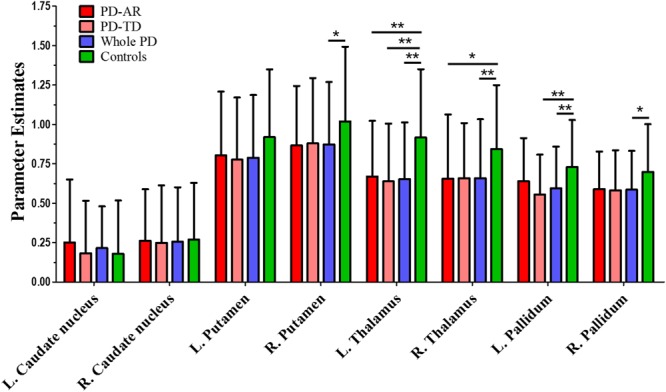
ROI-based analysis of parameter estimates identified by Independent Component Analysis algorithm. ^∗^*p* < 0.05; ^∗∗^*p* < 0.005.

In ROI-based analyses (**Figure 4**), similar findings were observed between whole PD group and controls or among the three groups. Decreased PE in the bilateral thalami (*p* < 0.001 and *p* = 0.004 for left and right, respectively) and pallidum (*p* = 0.004 and 0.011 for left and right, respectively) and right putamen (*p* = 0.037) was detected in whole PD patients in comparison with controls. In the PD subtypes, bilaterally decreased PE of the thalamus (*p* = 0.003 and 0.027 for left and right, respectively) was observed in the PD-AR patients and decreased PE of the left thalamus (*p* = 0.001) and left pallidum (*p* = 0.005) was observed in the PD-TD patients compared with controls. There was no significant difference of ROI-based PE between the PD subtypes.

### ROI-Based Inter-group Comparisons of ECM

**FIGURE 5:**
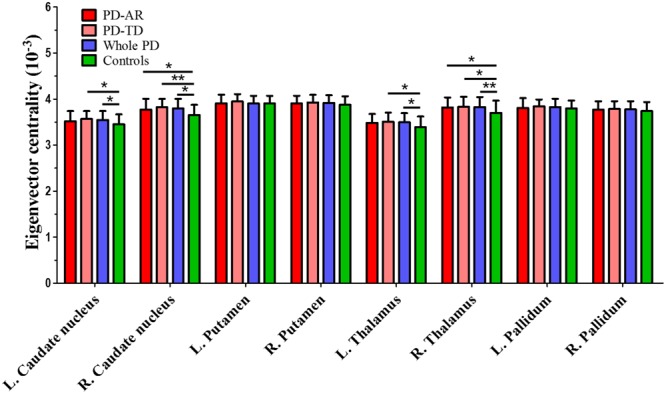
ROI-based analysis of eigenvector centrality identified by Eigenvector Centrality Mapping. ^∗^*p* < 0.05; ^∗∗^*p* < 0.005.

ROI-based ECM (**Figure 5**) was used to measure the connectivity degree and quality of each region within the predefined subcortical component of BGN. No decreased EC was observed between whole PD patients and controls or among the three groups. Instead, we found that significantly enhanced EC in the bilateral caudate nuclei (*p* = 0.015 and <0.001 for left and right, respectively) and thalami (*p* = 0.008 and 0.004 for left and right, respectively) in whole PD patients compared with controls. Enhanced EC in the right caudate nucleus (*p* = 0.014) and right thalamus (*p* = 0.029) in the PD-AR patients, and bilateral caudate nuclei (*p* = 0.031 and 0.001 for left and right, respectively) and bilateral thalami (*p* = 0.044 and 0.037 for left and right, respectively) in the PD-TD patients were observed in comparison with that in controls. No significant difference of EC was observed between the PD subtypes.

### Relationships between Brain Signal and Motor Impairments

We observed significant relationships of tremor and akinesia/rigidity with significantly reduced PE clusters in whole PD patients (**Figure 3B**). For occipital lobule, PE was positively correlated with tremor score (*r* = 0.319, *p* = 0.001) but negatively correlated with akinetic/rigid score (*r* = -0.378, *p* < 0.001). Similarly, for right cerebellum posterior lobule, we observed that the PE in this region was positive correlated with tremor score (*r* = 0.261, *p* = 0.008) and negatively correlated with akinetic/rigid score (*r* = -0.254, *p* = 0.010). No other cluster showed significant relationship between PE and clinical feature.

### Alterations of Functional Connectivity in Early PD

In a voxel-wised analysis, no significant voxel survived after AlphaSim correction (voxel *p* < 0.01, cluster size *p* < 0.05) between early PD patients and controls. Then ROI-based analyses were conducted in the both ICA data and ECM data. For ICA data, no significant difference was observed in the caudate nucleus (left, *p* = 0.642; right, *p* = 0.872), pallidum (left, *p* = 0.141; right, *p* = 0.412), putamen (left, *p* = 0.236; right, *p* = 0.266) and thalamus (left, *p* = 0.051; right, *p* = 0.227); for ECM data, there was no significant difference of EC in the caudate nucleus (left, *p* = 0.480; right, *p* = 0.143), pallidum (left, *p* = 0.520; right, *p* = 0.996), putamen (left, *p* = 0.677; right, *p* = 0.899) and thalamus (left, *p* = 0.314; right, *p* = 0.119).

## Discussion

Extensive connections of basal ganglia with cortex and limbic system (Ashby et al., 2010; Rolinski et al., 2016), and mutual structural connectivity between basal ganglia and cerebellum (Hoshi et al., 2005; Bostan et al., 2010) are commonly recognized. Through constructing BGN by ICA algorithm, we detected that basal ganglia diffusely coactivated with cortex, limbic system and cerebellum. By combing globally weighted functional connectivity within the subcortical component of BGN, we had three main findings: (1) compared with controls, whole PD patients and the PD subtypes showed decreases of PE within basal ganglia (e.g., pallidum and putamen) and thalamus; (2) compared with the PD-TD, significantly decreased functional connectivity between occipital lobule and cerebellum posterior lobule and basal ganglia was observed in the PD-AR, and such connectivity had positive correlations with tremor and negative correlations with akinesia/rigidity; (3) by calculating the ECM, we observed enhanced EC (enhanced global functional connectivity) in the caudate nucleus and thalamus in whole PD group and the subtypes compared with controls.

### Dysfunction of Functional Connectivity within BGN Measured by ICA

Parkinson’s disease is commonly attributed to the dysfunction of BGN, triggered by the nigral degeneration, with extensively connected brain regions involved (Braak et al., 2003). Therefore, these regions connected with basal ganglia, like precuneus and amygdala, were found to have reduced functional connectivity in whole PD group. Clinically, akinesia and rigidity are common to all PD patients while a proportion of them presents additionally moderate to severe tremor. Thus, PD is not a homogenous disease and could be divided into two dominant subtypes: PD-TD and PD-AR (Kang et al., 2005; Zaidel et al., 2009). It is generally believed that cortico-striatal-thalamic loop seems to be more close to akinesia/rigidity than in tremor (Pirker, 2003), while increasing evidence suggests that both striato-thalamo-cortical and cerebello-thalamo-cortical loops play a critical role in parkinsonian tremor (Helmich et al., 2011, 2012; Wu and Hallett, 2013). In the present study, the BGN identified by ICA covered both two loops and significant decreases of functional connectivity in the putamen, pallidum and thalamus in the PD subtypes compared with controls were observed. Supportably, previous documents reported reduced regional homogeneity (Wu et al., 2009) and functional connectivity (Szewczyk-Krolikowski et al., 2014; Rolinski et al., 2016) within the subcortical component of BGN in PD. Given that the dysfunction of functional connectivity in these subcortical component of BGN mainly influences the total motor impairment, the similar extent of motor disability (total UPDRS III score) in the PD subtypes probably explains the similar connectivity in these regions. Therefore, we supposed that disruption of the subcortical regions within BGN could be commonly seen in PD patients independent of tremor or akinesia/rigidity.

It was interesting to detect the changes in the functionally connected cortex by using ICA algorithm between the PD subtypes. We observed enhanced functional connectivity in the cerebellum posterior lobule and superior frontal lobule in PD-TD patients indicating increased connectivity of basal ganglia with cerebellum and frontal cortex. These findings, to a large extent, were consistently seen in previous studies (Helmich et al., 2011; Mure et al., 2011; Zhang et al., 2014; Chen et al., 2015). Cerebellum posterior lobule plays an important role in fine motor coordination. Compatible with Chen et al. (2015), we found significant increased connectivity between the cerebellum posterior lobule and basal ganglia which was positively correlated with tremor score but negatively correlated with akinetic/rigid score. However, researchers reported their findings were located in cerebellum anterior lobule (Mure et al., 2011). This discrepancy was probably attributed to the different methodology for the only regions having coactivity with basal ganglia were located in the bilateral cerebellum posterior lobules through ICA. In brief, to complement previous knowledge, our cerebellar finding indicated that functional connectivity of cerebellum posterior lobule with basal ganglia made an effort in modulation of parkinsonian motor impairments.

Moreover, reduced functional connectivity in the occipital lobule (visual cortex) was observed in the PD-AR patients compared with controls and the PD-TD patients. Weil et al. (2016) concluded in a review that patients with increased tremor perform better on vision-related tasks and the presence of tremor is associated with fewer color vision abnormalities. And, the association of visual deficits in PD-AR and relative sparing of visual dysfunction in PD-TD reflect distinct pathophysiological patterns of disease (Weil et al., 2016). Consistent with them, our finding of reduced functional connectivity of the occipital lobule with basal ganglia provided objective evidence for such different pathophysiological patterns. Moreover, PD-AR patients have a poorer prognosis and faster disease progression than PD-TD patients (Zaidel et al., 2009) for this specific subtype of PD would suffer from higher incidence of developing freezing of gait rather than PD-TD (Zhang et al., 2016). As occipital lobule is one of the main impaired brain region in PD patients with freezing of gait (Tessitore et al., 2012), negative correlation of functional connectivity in this region with akinetic/rigid score and positive correlation with tremor score further underscored its role in motor control and modulation. These findings updated previous documents that visual abnormalities were correlated with severity of gait impairment and freezing (Davidsdottir et al., 2005; Uc et al., 2005) and provided a new notion that disruption of coactivation between the occipital lobule and basal ganglia may be a functional substrate of akinesia and rigidity leading to poor prognosis.

### Enhanced Global Connectivity within the Subcortical Component of BGN Measured by ECM

Though the decreased functional connectivity within BGN, PD patients and the PD subtypes showed significantly enhanced EC in the caudate nucleus and thalamus compared with controls. EC attributes a value to each voxel in the brain such that a voxel receives a large value if it is strongly correlated with many other nodes that are themselves central within entire brain or parts of it (Lohmann et al., 2010). The enhanced EC in the caudate nucleus and thalamus indicates increased globally weighted connectivity. As previously discussed, reduced regional homogeneity and functional connectivity (Wu et al., 2009; Szewczyk-Krolikowski et al., 2014; Rolinski et al., 2016) within BGN was observed in PD patients, which was attributed to the impaired neuronal activity. However, PET studies consistently reported the existence of PD-related spatial covariance pattern with hyper metabolism in subcortex (e.g., thalamus) (Eidelberg et al., 1994; Eckert et al., 2007). To date, few study tried to explain such inconsistency. Through the combination of ICA algorithm and ECM analysis, we speculated that ICA algorithm aims to measure the oscillation of regional BGN influenced by PD while ECM quantifies the global connectivity (more close to the *global energy cost*) from each predefined region. Therefore, enhanced EC in the caudate nucleus and thalamus indicates up-regulation of activity from those survived neurons to balance the deficit of dopamine, which was supported by the hyper metabolism in these regions. Future studies would help solve this question by directly validating the relationship between EC and metabolism in normal controls and PD patients. Moreover, in the present study, we did not observe any difference of EC in the subcortical component of BGN between the PD subtypes. This result also supported the previous notion that similar alteration of subcortical functional connectivity characterizes PD patients with similar total motor disability in a global view regardless of motor dominant.

Some limitations had to be noted. First, we discarded mixed PD in the present study for this kind of patients had no symptom preference and the sample size is too small to reach a statistical result. Therefore, it should be cautious to translate present findings to mixed PD patients. Second, it is possible that lasting anti-parkinsonian management would reorganize and complement human brain function. Since more than 1/3 patients in the present study were taking medicine, medical influence on brain function would be inevitable though clinical assessments and image scanning were carried out after withholding all anti-parkinsonian medicine overnight. Third, imaging resolution of rsfMRI was relatively coarse, which would potentially influence our results. Nevertheless, the bigger PD database, compared with published documents, would enhance the robustness of our findings. Fourth, we did not detect any significant alteration of functional connectivity between the early PD patients and controls. We suspected that the main reason for the negative findings was the small sample size of the early PD patients. In the future, it deserves to validate the notion with enlarged sample size that whether dysfunction of BGN is an early biomarker for PD patients with acceptable sensitivity and specificity, which would obviously contribute to translating these findings into clinical trial.

## Conclusion

PD patients with similar total motor disability independent of motor subtypes consistently express similar alterations of functional connectivity (decreased within BGN but increased in a globally weighted view) in the subcortical component of BGN. Functional connectivity of cerebellum posterior lobule and occipital lobule with basal ganglia where differences were observed between PD subtypes play important roles in the motor modulation.

## Ethics Statement

This study was carried out in accordance with the recommendations of the Medical Ethic Committee of Second Affiliated Hospital of Zhejiang University School of Medicine with written informed consent from all subjects. All subjects gave written informed consent in accordance with the Declaration of Helsinki. The protocol was approved by the Medical Ethic Committee of Second Affiliated Hospital of Zhejiang University School of Medicine.

## Author Contributions

All of the coauthors listed meet the criteria for authorship. XG were involved with study concept and design, acquisition of data, analysis and interpretation of data, drafting/revising the manuscript. QZ, TG, JW, QG, and MX were involved with acquisition of data, analysis and interpretation of data. XX was involved with revising the manuscript. TW and PH were involved with analysis of data. MZ was involved with drafting/revising the manuscript and responsible for obtaining funding and supervision of study.

## Conflict of Interest Statement

The authors declare that the research was conducted in the absence of any commercial or financial relationships that could be construed as a potential conflict of interest.

## Footnotes

1 http://www.fil.ion.ucl.ac.uk/spm

2 http://rfmri.org/dpabi

3 http://mialab.mm.org

4 https://code.google.com/p/bias/source/browse/matlab/fastECM

5 http://fmri.wfubmc.edu/software/PickAtlas

6 http://marsbar.sourceforge.net

## References

[B1] Ashby F. G. Turner B. O. Horvitz J. C. (2010). Cortical and basal ganglia contributions to habit learning and automaticity. *Trends Cogn. Sci.* 14 208–215. 10.1016/j.tics.2010.02.001 20207189 PMC2862890

[B2] Bell A. J. Sejnowski T. J. (1995). An information-maximization approach to blind separation and blind deconvolution. *Neural Comput.* 7 1129–1159. 10.1162/neco.1995.7.6.1129 7584893

[B3] Bostan A. C. Dum R. P. Strick P. L. (2010). The basal ganglia communicate with the cerebellum. *Proc. Natl. Acad. Sci. U.S.A.* 107 8452–8456. 10.1073/pnas.1000496107 20404184 PMC2889518

[B4] Braak H. Del T. K. Rub U. de Vos R. A. Jansen S. E. Braak E. (2003). Staging of brain pathology related to sporadic Parkinson’s disease. *Neurobiol. Aging* 24 197–211. 10.1016/S0197-4580(02)00065-9 12498954

[B5] Chen H. M. Wang Z. J. Fang J. P. Gao L. Y. Ma L. Y. Wu T. (2015). Different patterns of spontaneous brain activity between tremor-dominant and postural instability/gait difficulty subtypes of Parkinson’s disease: a resting-state fMRI study. *CNS Neurosci. Ther.* 21 855–866. 10.1111/cns.12464 26387576 PMC6493074

[B6] Davidsdottir S. Cronin-Golomb A. Lee A. (2005). Visual and spatial symptoms in Parkinson’s disease. *Vision Res.* 45 1285–1296. 10.1016/j.visres.2004.11.006 15733961

[B7] Eckert T. Tang C. Eidelberg D. (2007). Assessment of the progression of Parkinson’s disease: a metabolic network approach. *Lancet Neurol.* 6 926–932. 10.1016/S1474-4422(07)70245-4 17884682 PMC2870718

[B8] Eidelberg D. Moeller J. R. Dhawan V. Spetsieris P. Takikawa S. Ishikawa T. (1994). The metabolic topography of Parkinsonism. *J. Cereb. Blood Flow Metab.* 14 783–801. 10.1038/jcbfm.1994.99 8063874

[B9] Guan X. Xuan M. Gu Q. Xu X. Huang P. Wang N. (2017). Influence of regional iron on the motor impairments of Parkinson’s disease: a quantitative susceptibility mapping study. *J. Magn. Reson. Imaging* 45 1335–1342. 10.1002/jmri.25434 27545971

[B10] Helmich R. C. Hallett M. Deuschl G. Toni I. Bloem B. R. (2012). Cerebral causes and consequences of parkinsonian resting tremor: a tale of two circuits? *Brain* 135(Pt 11) 3206–3226. 10.1093/brain/aws023 22382359 PMC3501966

[B11] Helmich R. C. Janssen M. J. Oyen W. J. Bloem B. R. Toni I. (2011). Pallidal dysfunction drives a cerebellothalamic circuit into Parkinson tremor. *Ann. Neurol.* 69 269–281. 10.1002/ana.22361 21387372

[B12] Hoshi E. Tremblay L. Feger J. Carras P. L. Strick P. L. (2005). The cerebellum communicates with the basal ganglia. *Nat. Neurosci.* 8 1491–1493. 10.1038/nn1544 16205719

[B13] Hughes A. J. Daniel S. E. Kilford L. Lees A. J. (1992). Accuracy of clinical diagnosis of idiopathic Parkinson’s disease: a clinico-pathological study of 100 cases. *J. Neurol. Neurosurg. Psychiatry* 55 181–184. 10.1136/jnnp.55.3.181 1564476 PMC1014720

[B14] Jin L. Wang J. Jin H. Fei G. Zhang Y. Chen W. (2012). Nigral iron deposition occurs across motor phenotypes of Parkinson’s disease. *Eur. J. Neurol.* 19 969–976. 10.1111/j.1468-1331.2011.03658.x 22288465

[B15] Kang G. A. Bronstein J. M. Masterman D. L. Redelings M. Crum J. A. Ritz B. (2005). Clinical characteristics in early Parkinson’s disease in a central California population-based study. *Mov. Disord.* 20 1133–1142. 10.1002/mds.20513 15954133 PMC3643967

[B16] Katzman R. Zhang M. Y. Ouang-Ya-Qu Wang Z. Y. Liu W. T. Yu E. (1988). A Chinese version of the mini-mental state examination; impact of illiteracy in a Shanghai dementia survey. *J. Clin. Epidemiol.* 41 971–978. 10.1016/0895-4356(88)90034-0 3193141

[B17] Lewis M. M. Du G. Sen S. Kawaguchi A. Truong Y. Lee S. (2011). Differential involvement of striato- and cerebello-thalamo-cortical pathways in tremor- and akinetic/rigid-predominant Parkinson’s disease. *Neuroscience* 177 230–239. 10.1016/j.neuroscience.2010.12.060 21211551 PMC3049982

[B18] Lohmann G. Margulies D. S. Horstmann A. Pleger B. Lepsien J. Goldhahn D. (2010). Eigenvector centrality mapping for analyzing connectivity patterns in fMRI data of the human brain. *PLOS ONE* 5:e10232. 10.1371/journal.pone.0010232 20436911 PMC2860504

[B19] Lou Y. Huang P. Li D. Cen Z. Wang B. Gao J. (2015). Altered brain network centrality in depressed Parkinson’s disease patients. *Mov. Disord.* 30 1777–1784. 10.1002/mds.26321 26180026

[B20] Luo C. Song W. Chen Q. Yang J. Gong Q. Shang H. F. (2017). White matter microstructure damage in tremor-dominant Parkinson’s disease patients. *Neuroradiology* 59 691–698. 10.1007/s00234-017-1846-7 28540401

[B21] Luo X. Qiu T. Jia Y. Huang P. Xu X. Yu X. (2016). Intrinsic functional connectivity alterations in cognitively intact elderly APOE epsilon4 carriers measured by eigenvector centrality mapping are related to cognition and CSF biomarkers: a preliminary study. *Brain Imaging Behav.* 10.1007/s11682-016-9600-z [Epub ahead of print]. 27714554

[B22] Mure H. Hirano S. Tang C. C. Isaias I. U. Antonini A. Ma Y. (2011). Parkinson’s disease tremor-related metabolic network: characterization, progression, and treatment effects. *Neuroimage* 54 1244–1253. 10.1016/j.neuroimage.2010.09.028 20851193 PMC2997135

[B23] Murphy K. Birn R. M. Handwerker D. A. Jones T. B. Bandettini P. A. (2009). The impact of global signal regression on resting state correlations: are anti-correlated networks introduced? *Neuroimage* 44 893–905. 10.1016/j.neuroimage.2008.09.036 18976716 PMC2750906

[B24] Obeso J. A. Rodriguez-Oroz M. C. Rodriguez M. Lanciego J. L. Artieda J. Gonzalo N. (2000). Pathophysiology of the basal ganglia in Parkinson’s disease. *Trends Neurosci.* 23(10 Suppl) S8–S19. 10.1016/S1471-1931(00)00028-8 11052215

[B25] Piccinin C. C. Campos L. S. Guimaraes R. P. Piovesana L. G. Dos S. M. Azevedo P. C. (2017). Differential pattern of cerebellar atrophy in tremor-predominant and akinetic/rigidity-predominant Parkinson’s disease. *Cerebellum* 16 623–628. 10.1007/s12311-016-0834-5 27853938

[B26] Pirker W. (2003). Correlation of dopamine transporter imaging with parkinsonian motor handicap: how close is it? *Mov. Disord.* 18(Suppl. 7) S43–S51. 10.1002/mds.10579 14531046

[B27] Post B. Merkus M. P. de Haan R. J. Speelman J. D. (2007). Prognostic factors for the progression of Parkinson’s disease: a systematic review. *Mov. Disord.* 22 1839–1851. 10.1002/mds.21537 17595026

[B28] Qiu T. Luo X. Shen Z. Huang P. Xu X. Zhou J. (2016). Disrupted brain network in progressive mild cognitive impairment measured by eigenvector centrality mapping is linked to cognition and cerebrospinal fluid biomarkers. *J. Alzheimers Dis.* 54 1483–1493. 10.3233/JAD-160403 27589525

[B29] Reinoso G. Allen J. J. Au W. L. Seah S. H. Tay K. Y. Tan L. C. (2015). Clinical evolution of Parkinson’s disease and prognostic factors affecting motor progression: 9-year follow-up study. *Eur. J. Neurol.* 22 457–463. 10.1111/ene.12476 24888502

[B30] Rolinski M. Griffanti L. Piccini P. Roussakis A. A. Szewczyk-Krolikowski K. Menke R. A. (2016). Basal ganglia dysfunction in idiopathic REM sleep behaviour disorder parallels that in early Parkinson’s disease. *Brain* 139(Pt 8) 2224–2234. 10.1093/brain/aww124 27297241 PMC4958897

[B31] Shen Z. Huang P. Wang C. Qian W. Yang Y. Zhang M. (2017). Increased network centrality as markers of relapse risk in nicotine-dependent individuals treated with varenicline. *Prog. Neuropsychopharmacol. Biol. Psychiatry* 75 142–147. 10.1016/j.pnpbp.2017.02.002 28185963

[B32] Smith S. M. Fox P. T. Miller K. L. Glahn D. C. Fox P. M. Mackay C. E. (2009). Correspondence of the brain’s functional architecture during activation and rest. *Proc. Natl. Acad. Sci. U.S.A.* 106 13040–13045. 10.1073/pnas.0905267106 19620724 PMC2722273

[B33] Szewczyk-Krolikowski K. Menke R. A. Rolinski M. Duff E. Salimi-Khorshidi G. Filippini N. (2014). Functional connectivity in the basal ganglia network differentiates PD patients from controls. *Neurology* 83 208–214. 10.1212/WNL.0000000000000592 24920856 PMC4117363

[B34] Tessitore A. Amboni M. Cirillo G. Corbo D. Picillo M. Russo A. (2012). Regional gray matter atrophy in patients with Parkinson disease and freezing of gait. *AJNR Am. J. Neuroradiol.* 33 1804–1809. 10.3174/ajnr.A3066 22538070 PMC7964749

[B35] Uc E. Y. Rizzo M. Anderson S. W. Qian S. Rodnitzky R. L. Dawson J. D. (2005). Visual dysfunction in Parkinson disease without dementia. *Neurology* 65 1907–1913. 10.1212/01.wnl.0000191565.11065.11 16282276

[B36] Weil R. S. Schrag A. E. Warren J. D. Crutch S. J. Lees A. J. Morris H. R. (2016). Visual dysfunction in Parkinson’s disease. *Brain* 5 102–106. 10.1093/brain/aww175 27412389 PMC5091042

[B37] Wink A. M. de Munck J. C. van der Werf Y. D. van den Heuvel O. A. Barkhof F. (2012). Fast eigenvector centrality mapping of voxel-wise connectivity in functional magnetic resonance imaging: implementation, validation, and interpretation. *Brain Connect.* 2 265–274. 10.1089/brain.2012.0087 23016836

[B38] Wu T. Hallett M. (2013). The cerebellum in Parkinson’s disease. *Brain* 136(Pt 3) 696–709. 10.1093/brain/aws360 23404337 PMC7273201

[B39] Wu T. Long X. Zang Y. Wang L. Hallett M. Li K. (2009). Regional homogeneity changes in patients with Parkinson’s disease. *Hum. Brain Mapp.* 30 1502–1510. 10.1002/hbm.20622 18649351 PMC6871162

[B40] Yan C. G. Wang X. D. Zuo X. N. Zang Y. F. (2016). DPABI: data processing & analysis for (Resting-State) brain imaging. *Neuroinformatics* 14 339–351. 10.1007/s12021-016-9299-4 27075850

[B41] Zaidel A. Arkadir D. Israel Z. Bergman H. (2009). Akineto-rigid vs. tremor syndromes in Parkinsonism. *Curr. Opin. Neurol.* 22 387–393. 10.1097/WCO.0b013e32832d9d67 19494773

[B42] Zhang D. Liu X. Chen J. Liu B. (2014). Distinguishing patients with Parkinson’s disease subtypes from normal controls based on functional network regional efficiencies. *PLOS ONE* 9:e115131. 10.1371/journal.pone.0115131 25531436 PMC4274088

[B43] Zhang H. Yin X. Ouyang Z. Chen J. Zhou S. Zhang C. (2016). A prospective study of freezing of gait with early Parkinson disease in Chinese patients. *Medicine* 95:e4056. 10.1097/MD.0000000000004056 27368041 PMC4937955

[B44] Zhang M. Y. Katzman R. Salmon D. Jin H. Cai G. J. Wang Z. Y. (1990). The prevalence of dementia and Alzheimer’s disease in Shanghai, China: impact of age, gender, and education. *Ann. Neurol.* 27 428–437. 10.1002/ana.410270412 2353798

[B45] Zhang Z. X. Roman G. C. Hong Z. Wu C. B. Qu Q. M. Huang J. B. (2005). Parkinson’s disease in China: prevalence in Beijing, Xian, and Shanghai. *Lancet* 365 595–597. 10.1016/S0140-6736(05)70801-1 15708103

